# Teamwork, Clinical Research, and the Development of Scientific Medicines in Interwar Britain: The “Glasgow School” Revisited

**DOI:** 10.1353/bhm.2007.0071

**Published:** 2007

**Authors:** Andrew J. Hull

**Keywords:** Glasgow, scientific medicine, academic medicine, clinician, medical scientist, teamwork, clinical research, laboratory, clinic, acidosis

## Abstract

This article argues that historians of medicine have, until very recently, misinterpreted the relationship of “science” and “the clinic” in the early twentieth century. It follows recent historiographic developments in focusing on the relationship in *practice* as exemplified by the development of a specific variety of collaborative clinical research using laboratory methods, ca. 1919–37, in a major British medical school. It suggests that it is such working hybrids that should be studied in order to understand fully the development of scientific medicines in the United Kingdom in this period. In Glasgow, it was the local medical culture’s characteristic local subservience to clinical priorities that facilitated, in a particular kind of academic unit, a certain type of hierarchical teamwork between clinicians and laboratory workers; the paper reveals how and why this teamwork became, over time, more of an equal partnership.

## Introduction

Until very recently, the historiography of the development of scientific medicine in Britain, and especially the evolution of academic clinical research in interwar hospitals, has had three main deficiencies. First, there was a residual implicit assumption of a fundamental essential binary opposition between “science” and “the clinic,” and this was compounded by an insufficiently critical translation of partisan early twentieth-century pro- and anti-medical-science rhetoric (which was part of an intraprofessional conflict) directly into the historical narrative.^1^ Second, this false dichotomy between science and the clinic was also reflected in a Whiggish overconcentration on the emergence of autonomous disciplines in the medical sciences, as if this teleology was necessarily implicit from the beginning of their development. Service roles were presented as producing only unnaturally constrained versions of laboratory science in Britain, while the range of interactions of science with the clinic was neglected.^2^ And third, the pro-academic-medicine rhetoric of state modernizers influenced by American models and money (like R. B. Haldane and George Newman) was also frequently accepted too uncritically. This often resulted in an unduly exclusive focus on the development of full-time academic units, and certain kinds of laboratory-influenced clinical research, which caused other (perhaps more typical) organizational groupings and types of research to be overlooked.^3^

However, the historiography has recently undergone a substantial evolution. Historians of medicine have begun to look at accommodations between science and the clinic, at teamwork, and at hybrid types of medical worker. They have also begun to stress the importance of location, and especially of the local medical culture, as a crucial factor informing the relationship between laboratory science and clinical practice, and to focus also on the development of local types of clinical research. The emerging consensus is that not one, but *many*, scientific medicine*s* evolved in Britain in the interwar period.^4^ In that vein, I will here explore the laboratory/clinic relationship in practice as it was shaped by, and shaped, the development of (a specific variety of) clinical research using laboratory methods in a major British medical school.

The question facing British medicine in the interwar period was, as Christopher Lawrence has argued, not whether a reformed, scientific medicine was imminent, but in what ways clinical medicine would change when it inevitably came.^5^ Laboratory scientists and clinical research workers had by now developed sufficient new institutional/financial power bases (via the Medical Research Council, the University Grants Commission, and the Rockefeller and Nuffield Foundations, which built new laboratories, funded new research, and facilitated the spread of academic medicine from universities to hospitals through full-time chairs) to rival the entrenched power of the clinical elite. Thomas Lewis, the figurehead for clinical research [Other P-570] using laboratory methods, seized this moment to go on the offensive for increased professional status and better career structures, pay, and facilities for such medical workers. In 1930, he deliberately provoked clinicians by asking, in the MRC’s annual report, the rhetorical questions: “Is there a Science of Experimental Medicine? Or is scientific work by the physician or the surgeon limited to the application in his art of scientific results worked out elsewhere in the laboratory and delivered to him for use?”^6^

Lewis’s forays fueled an increasingly hostile debate between clinicians and laboratory scientists (and their supporters) about who had the most effective clinical research methods. However, on the ground, in medical schools and teaching hospitals all over the United Kingdom (as well as at different teaching hospitals in London) these tensions had already been partially resolved and embodied in different local varieties of clinical practice and research using laboratory methods. How the roles of (mostly academic) clinicians and medical scientists interacted, overlapped, and changed over time in forming and reforming these emerging, evolving, and distinctive versions of clinical research, depended on the historical development of the local dynamic of medical power. This dynamic was embodied in the characteristic interactions of powerful local medical institutions that defined the local medical culture. I argue that it is crucial to understand these characteristic local interactions and their effects on practice and research to uncover the complex, heterogeneous interactions of science and the clinic in early twentieth-century Britain.

## Historiography of Glasgow Scientific Medicine

The historiography of scientific medicine in Glasgow long embodied the same essentialized opposition between clinic and laboratory that characterized the wider literature. Scientific medicine in Glasgow was portrayed as shaped by a number of key constraints: institutional circumstances led to clinical dominance, which led in turn to backward science and clinical research. How was this portrayal sustained?

N. D. Jewson’s pioneering historical sociology was fleshed out by the social historians of scientific medicine in the 1980s and 1990s noted above, who examined only what doctors *said* about the relationship between the clinic and the laboratory. Historians’ perceptions were partly shaped by Jewson’s typology of a chronological stage development of medical [Other P-571] knowledge/practice, and they thus identified in the partisan rhetoric not only an intellectual but also a professional, an economic, and a class battle.^7^ It was against this historiographic background that David Smith and Malcolm Nicolson, in the late 1980s, characterized the “Glasgow School.”^8^ They proposed a contemporary binary opposition between clinic and laboratory. The very title of their second 1989 paper spoke of “the clinic *versus* the laboratory.”^9^ They convincingly demonstrated that in Glasgow academic clinicians did cooperate on research with academic medical scientists, but the clinician was always the senior partner and set the research agenda and goals. Both types of worker openly disdained laboratory science as the primary medical authority, and attacked institutions in which it was so elevated.

However, Smith and Nicolson went further and also argued that this subservient relationship meant that Glasgow University’s laboratory science was backward, less progressive than at other universities, especially Cambridge: whereas F. G. Hopkins worked in an autonomous scientific discipline at the subprotoplasmic level on intermediate metabolism, Glasgow was stuck in an unproductive scientific dead end of old-fashioned protoplasmic theory with a gross, clinically oriented chemical physiology of ingestion, secretion, and excretion. Smith and Nicolson rather Whiggishly described Glasgow physiology as a “by this time somewhat outmoded tradition of scientific practice,”^10^ which was “isolated from the forefront of scientific advance and . . . vulnerable to clinical dominance” because of its characteristic “institutional circumstances.”^11^ This lamentable situation, they argued, was entirely due to the outcome of the local battle between science and the clinic in which the Glasgow School was, consistently and vocally, on the “clinicians’ side, in debates as to who the final arbiter of good medical knowledge was to be.”^12^ The key Glasgow triumvirate of the physiologists Diarmid Noël Paton and Edward Provan Cathcart and the clinician Leonard Findlay was characterized as enforcing an ancien régime [Other P-572] of the epistemological hegemony of clinical knowledge, which warped (in comparison with Cambridge) the local development of physiology (and thus of laboratory-influenced clinical research).

However, clinical dominance does not necessarily mean obsolete medical science. As Neil Morgan has pointed out, the study of large aggregates of protoplasm via colloid biochemistry was not perceived contemporaneously as an intellectual dead end or as backward science (unless one listens uncritically to the special pleading of Hopkins), but was itself a reductionist attempt to look at the chemical and thermodynamic function of complex chemical systems via the colloidal structural model of the membrane, and was thus “a natural part of the historical development of biochemistry, representing a conceptual bridge that linked the cell biology of the late nineteenth century with the era of structural macromolecular studies of proteins and nucleic acids that began after 1930.”^13^ In the period up to 1930, it provided a focus on a “legitimate problem” and “an alternative perspective to the metabolic enzyme programme.”^14^ Thus, while the Glasgow School’s commitment to the indivisibility of protoplasm may have been shaped by and shaped their reactionary social ideologies, it did not necessarily mean that they were practicing backward science.

Moreover, Smith and Nicolson’s argument—that the institutional circumstances in Glasgow equalled clinical dominance and backward science—circumscribed a seemingly inescapable stasis: it left no potential for change, for the dynamic evolution of the relationship of clinic and laboratory as individuals worked together. In addition, their evidence for the characterization of the Glasgow School was actors’ published statements;^15^ they did not explore how hospital-based clinical research was conducted in practice, and whether the relative role of clinic and laboratory were the same in reality as in the rhetoric, or changed over time. In Glasgow, by the start of the Second World War there was a full-time academic medical culture in many university-affiliated hospitals in which research groups led by clinical professors developed specialized clinical research programs using laboratory methods.^16^ In this paper, by a micro-analysis of teamwork in clinical research between clinicians and medical scientists, I show how this later kind of scientific medicine was facilitated by and was thus able to grow out of earlier developments.[Other P-573]

Smith and Nicolson were influenced by Gerald Geison’s arguments about the stagnancy of British physiology, and by Robert Kohler’s work on the development of biochemistry out of the medical chemistry part of physiology.^17^ Both linked intellectual with institutional developments, and Kohler pointed to the clinical service roles of medical science as important in facilitating its disciplinary development in Britain. However, this involved assuming an underlying teleological development in the form and content of knowledge, both in the formation of the discipline of physiology and in the birth of the discipline of biochemistry out of it. This led Kohler to the position that the British pattern of development exposed the institutional constraints that had warped the normal path of disciplinary evolution. Steve Sturdy has pointed out that the development of the medical sciences in Britain (because of, and not in spite of, such institutional factors) does not necessarily involve such linear disciplinary progress. He suggests that Medical Chemistry was a new kind of nondisciplinary scientific formation that allowed its workers to ignore “established boundaries, not just between scientific disciplines, but also between pure and applied science, and above all between laboratory science and the practice of medicine.”^18^ This suggests that a more flexible approach and a search for hybrid types of work and worker might yield a more nuanced understanding of scientific medicine in Britain.

Smith and Nicolson have more recently begun to suggest that the relations between clinic and laboratory were more fluid. Perhaps partly in response to such suggestions as Sturdy’s, they have proposed that some Glasgow practitioners, like Findlay (as well as Stockman), might be better characterized by the intermediate position of “clinician-scientist”: practitioners who combined a strong interest in laboratory science with a continuing commitment to clinical control of both practice and research.^19^ However, even this revision merely begs the question of what “clinician-scientists” did in practice, how they integrated science and the clinic in everyday patient care and research. How did the nature of that integration change over time? In order to get at this, it is necessary to look more [Other P-574] closely not just at what doctors *said*, but also at what they *did*; hence my focus here on clinical research.

More recent work on the evolution of scientific medicine elsewhere in Britain has confirmed the value of analyzing clinical research work, and has, as John Pickstone suggested, begun to focus on hybrids and team-work.^20^ Helen Valier, building on Sturdy’s mapping of the local “political economy” of scientific medicine in Sheffield, has begun to suggest how, in Manchester, there were many different ways of fostering many different kinds of scientific medicine in the interwar period.^21^ Sturdy argued that, in Sheffield, Arthur Hall gradually introduced one particular academic model of research based on full-time professional units into the hospitals. Valier argues that many types of organizational groupings of different kinds of medical scientists and clinicians were utilized by the reformist John Stopford (first as Manchester University’s medical dean, then as vice-chancellor) to foster scientific medicine, but that each interface had its own particular dynamics. One example, mainly working in (clinical applications of) pharmacology and physiology, was John Wilkinson’s Department of Clinical Investigations and Research, which included all the University’s scientific as well as clinical professors and which was eagerly supported by the Medical Research Council: this, Valier argues, was a “middle ground between the pre-clinical and clinical worlds . . . terrain to be shaped and maintained through whole-time clinical research.”^22^ I will argue that, in Glasgow, clinical research itself was a negotiated space, shaped by (and helping in turn to reshape) the local medical culture.

In Glasgow in this period, as Smith and Nicolson implied, clinical medicine so subsumed laboratory medicine that laboratory science was seen as an integral part of it. The research goals of this inclusive, but hierarchical, scientific clinical medicine were the better biochemical understanding of fundamental disease processes but also better, earlier diagnosis. Hospital-based academic research projects using laboratory techniques were framed around clinical problems. Thus clinical research conclusions meant immediately applicable lessons for clinical practice, although, initially, these would still be under the general oversight of the clinician. As [Other P-575] Smith and Nicolson noted, Paton’s low opinion of independent laboratory workers, even when he was professor of *physiology* at Glasgow University in 1906–28, was a matter of record.^23^ Teamwork thus achieves a new centrality in tracing the stories of scientific medicines in British medical schools and universities, rather than being important only when it embodies a particular kind of institutional or epistemological relationship between clinic and laboratory.

However, this is a story not simply of static institutional relationships and intellectual positions, but of shifting relationships and evolving positions. It was because of, and not in spite of, the close relationship of laboratory and clinic that laboratory methods and conclusions were eventually able to contribute more substantially to clinical decision-making in Glasgow. This is a story of an evolving concept of “laboratory science,” “the clinic,” and “clinical research,” and of the respective roles of the academic “laboratory scientist” and the “clinician” in one major British medical school, which, I argue, has implications for the way historians of medicine should approach the wider history of the relations between “science” and “the clinic” in interwar Britain.

## The Glasgow School Revisited

Sir Donald MacAlister (ex-clinician and chairman of the General Medical Council) was principal of Glasgow University from 1907 to 1929. Just like Hector Hetherington as principal nearly ten years later,^24^ MacAlister had his own strategy for nurturing the development of scientific medicine, in a period when applying science to medicine meant establishing new University lectureships in clinical scientific subjects with a base in the key voluntary teaching hospitals, and so helping to feed a culture of collaborative clinical research between clinicians and laboratory scientists. MacAlister, who had previously been a clinician in charge of child health at Addenbrooke’s Hospital and a member of the Cambridge University Medical Faculty, was alive to the difficulties of establishing cooperative relations between hospital and university—for example, the tensions underlined in recent attempts to install full-time academic units in some of the London teaching hospitals.^25^ He believed that he could achieve a measure of clinical research in some of the Glasgow hospitals via a limited number of medium-level full-time academic appointments, but [Other P-576] without destabilizing the existing part-time culture of the elite professor-clinicians who ran the academic departments. This policy itself built on previous developments. The first chairs that linked University science with the clinic came in Pathology. By 1893, the Western Infirmary (erected in 1874 as a purpose-built clinical resource for the University) boasted a joint University/hospital venture, the new Institute of Pathology that institutionally inscribed (and, thus, deepened) the very intimate relationship that had by then emerged between University preclinical science and hospital clinical practice.^26^

By ca. 1910, experimental and chemical physiology was the cutting edge of “science” to clinicians, and Glasgow University’s Physiology Department under Paton and his deputy Cathcart^27^ became the center of a talented group of workers who were to occupy key positions in physiological disciplines (and in university medical politics) in Glasgow and Scotland in the future. These included Walter Elliott, John Boyd Orr, Stephen Veitch Telfer, David P. Cuthbertson, Andrew Hunter, George Wishart, Leonard Findlay, Geoffrey Balmanno Fleming, Stanley Graham, Tom Honeyman, and Noah Morris.^28^ In a development that echoes that of Pathology and shows that biochemistry was now the must-have science for the clinic,^29^ in the mid-to-late 1920s three of these acolytes filled the three new posts of University Lecturer in Biochemistry/Clinical Biochemist at the three biggest Glasgow teaching hospitals: Telfer went to the Western Infirmary in 1925; Cuthbertson to the Royal Infirmary in 1926; and Morris to the Royal Hospital for Sick Children (RHSC) in January 1928, in the academic unit of Professor Leonard Findlay, whom he had already met when experimenting with Paton in the University Physiology Department. Findlay was a rich consulting clinician and classic social climber who affected the aristocratic manners of a bygone age, had an irascible temper, and ran his department autocratically—but he was also deeply interested in science. He had worked with Robert Muir in Pathology and [Other P-577] had an ongoing research relationship with Paton and Cathcart as part of the rickets project.^30^

The younger generation of Glasgow clinicians were by now trying to distinguish themselves from their lone-hero forebears by articulating a new culture of collaborative research.^31^ As Roger Cooter and Sturdy have argued, this kind of rational and efficient organization of medical resources had become a buzzword during the First World War, when, for example, the orthopedist Robert Jones and the cardiologist Thomas Lewis, both deeply interested in physiological function, had sought and won state support for fast-acting, money-saving programs of rehabilitation for military personnel. Jones, in his organization of orthopedic centers, and Bertrand Dawson—author of the 1920 Dawson report on the reorganization of British medical services—had also proposed rational, hierarchical teamwork maps of the division of labor between different medical workers in different locations. These maps echoed the hierarchical organization of military medical services on the Western Front (from casualty clearing station to base hospital at home) and spoke of the increasing militarization of British medicine and society. George Newman was also an outspoken supporter of medical team-work, and the division and cooperation of medical labor became important criteria in securing MRC and other grant monies.^32^ David Smith has shown how at the Rowett Research Institute of Animal Nutrition in Aberdeen during the 1920s and early 1930s one product of the Glasgow School, Boyd Orr, consistently utilized a hierarchical version of teamwork to constrain research roles and the research agenda to ensure that his practical, applied concerns in animal nutrition were never superseded by the more academic, scientific, pure-research concerns of his laboratory staff.^33^

Findlay strongly supported a similar version of hierarchical teamwork that underlined the primacy of clinical applications. He did much of his experimental work on rickets in Paton’s physiological laboratories (himself experimenting on dogs), and together they undertook a combined clinical and experimental study of tetania parathyreopriva. As Findlay wrote in 1922:[Other P-578]

This co-partnership, which I particularly prize, has continued till the present time. In this way the more or less academic aspect of disease has been brought into intimate association with the clinical, and vice versa, to the great benefit of both sets of workers, and . . . to the greater benefit of medical research than would have occurred had the two types of workers remained independent. A young physician or a young surgeon during his waiting period not uncommonly spends some time in a laboratory, but it is exceptional for the head of a physiological department and the head of a clinical department to become allied for serious and consecutive research. In my opinion the ideal would be to extend such a combine to include the chiefs of a pathological department and of a surgical department, so that as comprehensive an idea of vital phenomena as possible would be obtained.^34^

In fact, Findlay even contributed to the London debate on the nature, scope, and control of clinical research in October 1930. His contribution makes clear the kind of hierarchical teamwork in clinical research that was possible in Glasgow, and why. Speaking after John Ryle at a Royal Society of Medicine discussion on research in medicine, he struck the keynote of the relationship between the laboratory and the clinic in contemporary Glasgow when he “declared himself unable to understand the attempt to distinguish between the research worker and the clinician. *Laboratory work was still clinical medicine*.”^35^ In Glasgow, laboratory sciences were not there just to serve clinical medicine, they were subsumed within it. In his own academic unit, Findlay put this Glaswegian vision of clinical research into effect. It was eventually to foster a rather different relationship between science and the clinic from the one he favored, as ongoing collaborative research shaped science/clinic relations.

## Changing Conceptions of Teamwork at the Royal Hospital for Sick Children Group (Yorkhill, Glasgow)

Glasgow’s RHSC was a voluntary hospital originally founded in 1882 which had moved to a purpose-built building on a new site on the Yorkhill estate, on raised ground that afforded fine views across the River Kelvin to the Western Infirmary and the University on Gilmorehill, just before the outbreak of the First World War, in July 1914.^36^ The hospital’s Board of Management [Other P-579] had a strong and progressive policy of encouraging the development of new specialties and scientific research. Both Robert Barclay, the enterprising chairman of the hospital’s Board of Directors, and University Principal Donald MacAlister, chairman of the governors, vocally supported this policy, as did Cathcart (who served on the BOM from 1933 to 1936 as representative of the University Court, and thereafter as Senate representative) and Findlay. MacAlister argued in 1923 that a modern teaching hospital “must undertake research as well as instruction, and give assistance and facilities to its staff for scientific investigation which might benefit mankind.”^37^ Furthermore, MacAlister, Cathcart, and Findlay ensured leverage in the Medical Faculty and at the University Court to obtain University money as well as private donations to make these plans a reality.^38^

In the Department of Medical Paediatrics, a group of academic clinicians and laboratory scientists was put together by MacAlister and run by Findlay (and then by Geoffrey Balmanno Fleming) with joint hospital, University, and MRC financial support as a very special kind of academic unit that practiced collaborative clinical research. They were a team, and all brought their own skills to bear on clinical problems; Findlay always listed the pathologist and the biochemist as well as the clinicians when speaking of his staff.^39^

The relationship between the University and the RHSC was already close: in 1919 a Lectureship in Orthopaedics (children) had been established with funds from Robert Barclay, and one on the Medical Diseases of Infancy and Childhood with money from Leonard Gow. In 1924, a Chair of Medical Paediatrics was established with a bequest from the late William Gemmell, in memory of his brother, Dr. Samson Gemmell, formerly Regius Professor of Medicine in the University. The principal and the RHSC directors had actively courted all of these links.^40^ They finally completed the academic line-up (what the directors called the hospital’s “team of scientific workers associated with the University”)^41^ needed to begin an intensive research program on the diseases of childhood, and [Other P-580] particularly on the scourge of rickets,^42^ with the appointment of Dr. John Blacklock to the post of University Lecturer in Pathology in October 1928.^43^ As with Noah Morris’s January 1928 appointment, for which he personally carved the money out of University funds,^44^ Principal MacAlister was again closely involved in the establishment of the new lectureship.^45^ Also supportive were Professors Muir (Pathology—who recommended Blacklock), Paton, Cathcart, and Findlay.^46^

Only two months later, the directors sent a memorandum to all the members of the Department of Medical Paediatrics (that is, to the two visiting physicians—the Professor of Medical Paediatrics, Findlay, and the Lecturer on the Medical Diseases of Infancy and Childhood, Fleming—and to the biochemist, Morris, and the pathologist, Blacklock). The memorandum urged them all to coordinate the teaching they were required to do, and reminded Morris and Blacklock of the stipulation in their contracts that, as well as teaching and routine service work (tests), they must cooperate in collaborative research with the clinicians. Morris’s contract described part of his duties as

promoting the advancement of medical knowledge in relation to Infancy and Childhood by scientific investigation; and assisting the Physicians and Surgeons by undertaking chemical examinations and tests in relation to the patients under their charge, and carrying out within the Hospital, clinical observations and investigations on their behalf or at their instance.^47^

The memorandum went on to make clear that the hospital had assembled this very special and well-staffed academic department piece by piece for a very specific purpose: to fulfil Barclay’s, MacAlister’s, Cathcart’s, Paton’s, and Findlay’s goals of medical and surgical advance through collaborative clinical research:[Other P-581]

the Directors suggest the advantage of the . . . Professor and Lecturer and the Pathologist and Biochemist coming to an arrangement for co-operation in research work *without any subordination of the one to the other* each having independent University and Hospital appointments, and that they along with the Radiologist . . . might form a Medical Research Department of which said Professor would naturally be the Head and said Lecturer his deputy. In the matter both of research and treatment, the Directors feel that they cannot too strongly urge the advantage of the fullest co-operation on the part of all the members of the Hospital and Dispensary Staff Medical and Surgical and Specialists. Only thus will the members of the Staff be enabled to utilise all the facilities provided by the Directors, and develop fully their own Departments. The Directors desire and request that the Visiting Physicians and Surgeons will give all reasonable facilities to the Pathologist and Bio-Chemist in connection with the special research work of their particular Departments. The Medical Laboratory shall be available for the use of the Visiting Physicians, the Assistant Visiting Physicians, and others.^48^

Morris, who became the first University Lecturer in Pathological Biochemistry (and Biochemist) at the RHSC in 1928, continued to have charge of a clinical ward until 1930, when the supervision of research work in the Biochemical Department had begun to take up so much of his time that he was forced to relinquish it. However, as the testimonials for his later chair note, he “continued . . . to be closely associated with the clinical work of the hospital”:

I make regular ward rounds with the visiting physicians (Professor G. B. Fleming and Dr Stanley Graham) and with one of the visiting surgeons (Mr Matthew White). Under their general direction I have control of certain beds for patients suffering from disorders of metabolism and take an active part in the treatment of patients, especially those with disorders of biochemical interest. On the surgical side I am associated with Mr White in the investigation of certain aspects of general treatment and at the present time we are undertaking investigations into the healing of fractures, treatment of burns and the effect of various forms of anaesthesia. I assist Dr Graham in the supervision and after-care of patients with celiac disease, and with Professor Fleming am actively interested in following up the effects of various forms of treatment in patients with dental caries. In 1928 I instituted an out-patient clinic for diabetic patients, the first in Glasgow: this, which is still being conducted under my personal supervision has proved of very great help in the treatment of the diabetic patient and has already yielded information of considerable value which has been published in the medical press.^49^

Medical Research Council personal grants and expenses from at least 1926 to 1945 allowed the group to pursue collaborative research that nearly all fell within the Paton/Findlay/Cathcart rickets project.^50^ Most of the work, as Smith and Nicolson have argued, consisted of gross metabolic studies within the intellectual remit of physiological chemistry—analysis of the body’s excretion of nitrogenous compounds, and analysis of the blood and tissues to determine the levels of metabolites in disease. Generally, the research work at the RHSC was directed toward finding accurate biochemical aids to earlier diagnosis; however, over time, a distinct evolution can be detected in the goals of the research, and particularly in the relative positions of laboratory and clinic.

Morris’s three earliest, pre-RHSC research papers, written when he was in the Physiology Department, reflect the classic Glasgow School agenda: a clinical observation is used as the basis of a piece of research that seeks merely to use laboratory methods to confirm the correctness of the existing interpretation of the clinical sign. His third paper, “The Cause of Increased Electrical Excitability” (1922), even ends by citing some of Findlay and Margaret Ferguson’s work on the socioeconomic causes of tetany/rickets (poor ventilation in overcrowded tenements).^51^ Once he had joined the RHSC group, Morris’s research became collaborative. Typically the clinician Stanley Graham closely cooperated with Morris the biochemist (who either had his own ward or freely used Graham’s or Findlay’s patients).^52^ For example, in their 1928 paper “The Regulation of the Acid-Base Balance of the Body” Morris and Graham follow their usual practice of literally beginning with a stated clinical observation and then going on to confirm it (or, one could say, reconceptualize it) scientifically. They seek to sharpen clinical acumen by providing clinicians with simple laboratory-derived tools (biochemical tests), which will consolidate clinical observation. They stress that the presence of ketone bodies in urine is not diagnostic of acidosis, nor are estimations of the alkali reserve (an estimate of total CO_2_—combined and free—in blood) indicative:

Taken apart from other determinations and clinical observations, the figure for the alkali reserve may be dangerously misleading. . . . The reaction of the blood is maintained as constant as possible by a continual variation of the respective [Other P-583] values of . . . several factors, so that an estimation of the value of any one, or any particular clinical symptom, such as whether alkaline reserve, free CO_2_ rapid breathing, may be wholly misleading. It is only by a combination of various findings, chemical and clinical, that one can arrive at any conclusion.^53^

Morris and Graham, a biochemist and a clinician working together, sought to put clinical diagnosis on a scientific basis—to found it on an investigation of the causal factors of disease that routinely used laboratory methods. Their conclusion is typical of the Glasgow School: the laboratory *alone* is not a reliable guide to clinical practice.

However, this and other papers from 1927–30 also represent a slight alteration in the goal of research, and in the implied relative positions of laboratory and clinic, from Morris’s earliest papers.^54^ There has been a slight but discernible recalibration of the clinic/laboratory relationship: it is argued that laboratory methods are now a *necessary*, though not a *sufficient*, guide to clinical practice. The laboratory is still serving the clinic, but it is now recognized as making a uniquely valuable contribution to diagnosis. No longer is medical science just *confirming* clinical observations, it is also sometimes *guiding* them as a more equal partner would. This interpretation is confirmed by Findlay’s revealing opening contribution to the discussion that ensued when this paper was first delivered to the Glasgow Royal Medico-Chirurgical Society:

Dr. Leonard Findlay said he felt a certain diffidence in taking part in the discussion, which savoured somewhat of the highest mathematics, but that he would like to say some words regarding the work from the clinical point of view. These words might have a special interest, coming as they do from the mouth of the clinician in whose department a great deal of the work has been done. First of all, there is a consolation in the assurance that the lecturers have given us that there is no fear of us clinicians being asked to pursue this work as a routine measure. This is certainly comforting to those of us who are not so [Other P-584] young as we once were and are becoming less receptive of the newer clinical methods. This does not, of course, mean to say that this type of investigation should not be done. In fact, in view of our ignorance of the subject and its clinical application it is exceedingly important . . . that investigation of certain well-defined diseases be carried out, so as to reveal to us the changes which occur in the acid-base balance in these diseases and perhaps give us a key to their explanation.^55^

Findlay then summed up his general view of this kind of work: “As a clinician I should say that the greatest benefit that one has derived from living alongside work like this is that one’s clinical acumen has been stimulated.”^56^ He went on to list the exact clinical observations that corresponded to the states that Morris and Graham had described biochemically, and stressed that “as *physicians* we must be able to recognise these conditions if we are to apply any appropriate remedy.”^57^ He immediately needed to reclassify/reclaim their work as part of the clinical project: the normal methods of investigation of the clinician. Notice that he said that the penetration of the clinical gaze had been *stimulated*, not *improved*, by this work. Findlay’s position was akin to that of Lawrence’s late nineteenth-century elite physicians who objected to relying solely on the reading given by Clifford Allbutt’s new more clinic-friendly thermometer as arbiter of diagnosis and treatment: they maintained, as Lawrence argues, that “it only gives a piece of information to be concocted by the clinical art.”^58^ Findlay, a type of “clinician-scientist” more clinical than scientific, jealously defended the primacy of his own inductive methods from the encroachments of laboratory science. He claimed to be a better clinical researcher than the biochemist and scientific clinician, even if his expertise was largely of the supervisory type—marshaling, weighing, and judging different types of evidence. Findlay was reasserting the old Glasgow School clinic/laboratory nexus, but it was clear that this traditional relationship was already under threat from endogenous change.

Clearly Morris and Graham had been socialized by their experiences at the hospital into accepting this view of the relative roles of the clinician and the scientist. Graham replied to Findlay regretting that “although we have been engaged in this sort of investigation for the past few years, we do not think that, at present, much help has been given to the clinician”—but the *reason* he gives for this (“mainly because of the difficulty of the laboratory methods involved”) indicates that they are beginning [Other P-585] to shift away from Findlay’s position.^59^ This work was expanded and published in book form in 1933 as *Acidosis and Alkalosis*; it was intended as a simple potted guide for clinicians to a new scientific style of diagnosis so that, as the authors explained in their preface, “the knowledge acquired in the laboratory can now with profit be employed at the bedside.”^60^ In fact, what they also meant was that the results of collaborative clinical research could be translated into guidelines for clinical practice.

However, when Morris rewrote this work for his D.Sc. thesis in 1935, he put a much more radical spin on it in which he saw the results of collaborative clinical research as embodying much firmer rules of engagement for the lone clinician. He now argued that clinicians, unguided by laboratory scientists, had begun to see acidosis/alkalosis everywhere. Morris felt it his scientific duty to correct their overenthusiastic, unscientific use of these diagnostic labels:

There are three stages in the evolution of any scientific advance. The first is a period in which it is either entirely neglected or regarded with too rigorous a scepticism. The second stage is one in which the over-sceptical attitude is followed by an equally exaggerated credulity. Finally comes the period in which it is estimated at its proper value. At the present time the clinical conception of acidosis is in the second phase with the result that many conditions are cheerfully labelled acidosis. No state from debility to rheumatism is safe from this diagnosis and this is especially true for infancy and childhood. So much is this the case that the scientific foundation on which is based the conception of acidosis is likely to be lost sight of and ignored. This would be a loss both from the practical and scientific aspects of medicine, since the elucidation of the problems of acidosis has thrown considerable light on many aspects of clinical medicine and has led to the adoption of valuable therapeutic measures.^61^

Morris ended this introduction arguing that certain diagnosis even of the sign of these conditions can be achieved only by the triangulation of clinical with laboratory work—with each given equal weight:^62^

There is no royal road to diagnosis of acidosis or alkalosis. There is no single clinical sign or chemical test which is pathognomic of either condition. . . . It is therefore only by a consideration of all the evidence clinical and laboratory that one can come to any definite conclusion. In many diseases unfortunately, the knowledge of the nature of the acid-base disturbance is still so scanty, that [Other P-586] such a procedure is likely to lead to fallacious conclusions. Before the various tests can be used with any degree of safety it is necessary that a thorough investigation be made of the various manifestations clinical and biochemical of the acid-base disturbance in these conditions. In a number of diseased conditions, however, the clinical and biochemical findings have been correlated so that it is possible now to label them acidosis or alkalosis.^63^

Morris was now arguing, therefore, that collaborative research between clinicians and laboratory scientists (of which he was, of course, both) should be conducted on a much more equal footing. However, in warning that unguided clinicians were wont to mistake the superficial diagnosis acidosis as identification of the underlying disease when, in fact, it was no more than a sign, he was also arguing that clinicians needed to take much greater heed of laboratory-derived evidence in their clinical practice.

In redefining the relationship between clinician and laboratory worker in this way, Morris still echoed his early teacher in physiology, Paton, but a different aspect of Paton’s thoughts on this relationship. Much of the time, Paton believed that medical scientists worked without reference to clinical problems and priorities; it was not surprising, therefore, that their work was of limited clinical usefulness. However, doing clinically relevant work did not necessarily mean dancing entirely to the clinician’s tune. Paton thought there was also a need for intermediary, hybrid medical workers who, though physiologists, were able to translate laboratory work into the clinical context, rather in the same manner as Thomas Lewis’s research physicians. In an unusually candid article in the volume *Physiology and National Needs* (and perhaps his forthright tone was because the importance of the medical science was for once the focus), he reversed the attitude embodied in the above quotation to argue that “without a knowledge of physiology the physician is merely groping in the dark” because the earliest manifestations of disease were functional disturbances, not structural lesions.^64^ On this basis, he argued that

the hospital physician . . . must keep in touch with the growing science of physiology, and must welcome all applications of physiological methods to his work. What is wanted is some one who can show them how these scientific methods are to be carried into practice at the bedside, some one who will keep before the students the necessity of applying physiological knowledge to find out what is different from the normal action in every case—in fact, a Clinical Physiologist. . . . It is the man trained as a physiologist who is prepared to devote his [Other P-587] whole time to the study of disease in the wards of the hospital, who is of real use in advancing knowledge and in training students to carry on such work.^65^

Morris’s discussion of acidosis also revealed his commitment to medical holism.^66^ This had long supported the characteristic Glaswegian relationship between laboratory and clinic, but Morris now gave it a new scientific twist. For Findlay, a holistic appreciation of disease was one of the factors that made the clinician’s skills of prime importance in the practice of medicine and in the advance of medical knowledge.^67^ For Morris, as for Paton, a functional, holistic understanding of disease was the optimal way to integrate the skills of science with those of the clinic. The body was a complicated biostatic system; the physician’s job was to help the body to restore equilibrium.^68^ What seemed like symptoms were often attempts to restore normal functioning. Only a scientific clinician with an understanding of the deep physiological processes of the body’s integrated self-regulation could correctly diagnose using a range of evidence, and intervene effectively by making conditions as favorable as possible, thus allowing the *vis medicatrix naturae* to rebalance the body into health.^69^ It was for this reason that the doctor should *combine* the laboratory research methods of Paul Ehrlich and the patient observational science of Hippocrates.^70^

By 1936–37, Morris had begun research on enzymes.^71^ This could indicate a broadening of his scientific agenda from chemical physiology to a more Hopkinsian conception of biochemistry, but it did not mean the abandonment of the new, more equal partnership between laboratory and clinic in favor of the epistemological dominance of the laboratory promoted by Hopkins. It would be the *next* transitional generation that [Other P-588] precipitated the arrival in Glasgow of biomedical disciplines more (but, crucially, never completely) independent of clinical medicine. In 1947 J.N. Davidson was appointed to the Chair of Physiological Chemistry. He specifically cited the example of Hopkins’s Dunn Institute of Biochemistry at Cambridge as the model that Glasgow should follow.^72^

Morris was willing to theorize that a biochemical test could be more clinically useful than clinical observation, but he concluded from his laboratory observations that, in the case of phosphatase, it was not. In December 1936 he characteristically argued that “the estimation of plasma phosphatase has a place in the diagnosis of the pre-clinical stage of rickets, but cannot by itself be used to indicate *in the individual patient* either the severity of the rachitic process or the presence or absence of healing.”^73^ Yet in the very first sentence of his first 1937 paper on phosphatase, he stated explicitly that his work was geared toward establishing whether the presence of increased quantities of the enzyme in the blood was an independent diagnostic index for rickets:

Although it is true that ordinary clinical examination suffices for the diagnosis of moderate degrees of rickets, most workers would agree that *something more is necessary* for the detection of milder forms of the disease, when the *metabolic disturbances* are not sufficiently great or have not continued long enough to produce clinical manifestations.^74^

But by later in 1937 he had concluded, finally, that, at least in bone disease, “It is clear that the determination of plasma phosphatase by itself is of little value in either diagnosis or prognosis. *Taken in conjunction* with clinical, radiological, and other biochemical findings it may yield considerable assistance.”^75^

Certainly Morris had shifted from studying the gross metabolism of chemical physiology to studying the intermediate metabolism of biochemistry, and this shows that he was alive to contemporary scientific developments. However, though he was clearly perfectly willing to search for and evaluate biochemical tests that might outperform clinical judgment, he was not at all dogmatic about insisting on the primacy of laboratory-derived [Other P-589] knowledge. The final arbiter was still the clinical usefulness. He was, thus, clearly not endorsing the Hopkinsian scientific project, but had, rather, transferred his modified clinical agenda (in which clinical and laboratory findings had an *equal* value) to support his work within biochemistry. He thus maintained a distinctive, revised version of the traditional Glasgow School position on the relationship of the laboratory and the clinic: equally valid partners.

## Conclusion

University Principal MacAlister and his reformist allies in the University of Glasgow Medical Faculty pursued an alternative strategy to the full-time professorial unit and created a collaborative academic clinical research unit at the RHSC with University, hospital, and MRC funds, *without* disturbing the existing part-time culture of clinicians. The unit was not hampered by, but thrived on, the clinical cast of Glasgow medicine. This local medical culture was not anti-laboratory-science but rather supported a characteristic way of accommodating science in clinical research and practice that blurred the boundaries between the two. For Findlay, clinical acumen in bedside practice was always deepening its penetrative gaze by adopting the latest diagnostic technique (instrument or biochemical test).^76^ At the same time, in research, the laboratory worker and the clinician worked together as part of the same enterprise. For Findlay, both were subsumed within clinical medicine, which itself included and linked both clinical research and practice. The clinician still held intellectual primacy and dictated the research agenda: clinically observed problems with the goal of better, earlier clinical diagnosis. Morris and his co-workers were socialized into this local culture of medical knowledge, which had its roots in the historical power relationships between local institutions. However, Morris’s own conception of the relative roles of clinician and laboratory worker was forged in Paton’s physiology laboratory, and he soon evolved into a Patonian “Clinical Physiologist,” bringing a new physiological dimension into the older Glasgow clinical holism.

Thus did laboratory and clinic interact in an old British clinical medical stronghold to produce a characteristic and evolving local version of scientific medicine, in which the final clinical application was still the key medical moment, but which was informed more equally by both clinical and laboratory inputs. The meaning of scientific medicine was a locally [Other P-590] negotiated, dynamic process. The institutional circumstances in Glasgow therefore do not, contra Smith and Nicolson, necessarily embody a static view of either medical science or the relationship between that science and the clinic. Rather, it was this revised, physiologically reanimated, conception of Glasgow clinical research and practice with its functionalist holism that Morris took with him to the full-time Chair of Materia Medica and Therapeutics at Stobhill. As he noted in his inaugural address:

If . . . therapeutics is to have a scientific foundation, it is essential that every effort should be made to obtain a conception of disease founded on the advances of modern science applied to medicine. *Clinical observation is not enough*.^77^

Tracing the evolution of scientific medicine in Glasgow has thus revealed, in the teamwork of laboratory and clinical workers, a constantly evolving relationship between “science” and “the clinic” that underlines the importance of such local studies for understanding the development of scientific medicines in Britain in the interwar period. Different kinds of laboratory science in different places interacted in different ways with clinical medicine, to produce a series of new local varieties of scientific medicine: new local versions of clinical research and practice. There were also many different institutional formations—differently assembled, affiliated, and funded teams—which produced this variety of styles of scientific medicine. Fundamental in shaping this process in each location was the local medical culture. Thus, in studying the development of scientific medicines in Britain in the interwar period, historians of medicine should focus not only on the general, the national, but also on the particular, the local. They should be mindful of the evolution of a dynamic process, with feedback loops, and should not be bound by constraining static definitions of institutional circumstances, local medical cultures, “science,” or “the clinic.” They should look for hybrids, interactions, and teamwork rather than opposition and division, and should examine a range of institutional groupings, not just full-time academic units.[Other P-591]

## Footnotes

1
	    See, e.g., LawrenceChristopher“Incommunicable Knowledge: Science, Technology and the Clinical Art in Britain, 1850–1914,”J. Contemp. Hist.198520503201161728910.1177/002200948502000402 Lawrence“Still Incommunicable: Clinical Holists and Medical Knowledge in Interwar Britain,”Greater than the Parts: Holism in Biomedicine, 1920–1950LawrenceChristopherWeiszGeorgeOxfordOxford University Press199894113 JacynaL. S.“The Laboratory and the Clinic: The Impact of Pathology on Surgical Diagnosis in the Glasgow Western Infirmary, 1875–1910,”Bull. Hist. Med.1988623844063067788 . American work has also been influential on British historiography: see GeisonGerald L.“Divided We Stand: Physiologists and Clinicians in the American Context,”The Therapeutic RevolutionVogelMorris J.RosenbergCharles E.PhiladelphiaUniversity of Pennsylvania Press19796790 MaulitzRussell C.“‘Physician Versus Bacteriologist’: The Ideology of Science in Clinical Medicine,”ibid.91107
	    
	

2
	    See, inter alia, GeisonGerald L.“Social and Institutional Factors in the Stagnancy of English Physiology, 1840–1870,”Bull. Hist. Med.19724630584552626 GeisonMichael Foster and the Cambridge School of Physiology: The Scientific Enterprise in Late Victorian SocietyPrincetonPrinceton University Press1978 RomanoTerrieMaking Medicine Scientific: John Burdon Sanderson and the Culture of Victorian ScienceBaltimoreJohns Hopkins University Press2002 KohlerRobert E.From Medical Chemistry to Biochemistry: The Making of a Biomedical DisciplineCambridgeCambridge University Press1982 ColemanWilliamHolmesFrederic L.The Investigative Enterprise: Experimental Physiology in Nineteenth-Century MedicineBerkeleyUniversity of California Press1988
	    
	

3
	    See, e.g., BynumW. F.“Sir George Newman and the American Way,”The History of Medical Education in BritainNuttonVivianPorterRoyAmsterdamClio Medica199537507671593 GrahamGeorge“The Formation of the Medical and Surgical Professorial Units in the London Teaching Hospitals,”Ann. Sci.1970261221160955010.1080/00033797000203377 FisherDonald“The Rockefeller Foundation and the Development of Scientific Medicine in Great Britain,”Minerva19781620411161066210.1007/BF01102180 SturdySteve“The Political Economy of Scientific Medicine: Science, Education and the Transformation of Medical Practice in Sheffield, 1890–1922,”Med. Hist.19923612559158397210.1017/s0025727300054983PMC1036558 SturdySteveCooterRoger“Science, Scientific Management, and the Transformation of Medicine in Britain c. 1870–1950,”Hist. Sci.199836421661162055410.1177/007327539803600402 . See also LawrenceChristopher“Clinical Research,”Science in the Twentieth CenturyKrigeJohnPestreDominiqueAmsterdamHarwood Academic Publishers199743960
	    
	

4
	    See LawrenceChristopher“A Tale of Two Sciences: Bench and Bedside in Twentieth-Century Britain,”Med. Hist.199943421491088514310.1017/s0025727300065686PMC1044179 but more especially the following: ValierHelen“The Politics of Scientific Medicine in Manchester, c. 1900–1960” Ph.D. thesis University of Manchester2002 LawrenceChristopherRockefeller Money, The Laboratory, and Medicine in Edinburgh, 1919–1930Rochester, N.Y.University of Rochester Press2005 . See also WeatherallMarkGentlemen, Scientists and Doctors: Medicine at Cambridge 1800–1940Woodbridge, U.K.Boydell Press2000142311 SmithDavid“The Use of ‘Team Work’ in the Practical Management of Research in the Inter-War Period: John Boyd Orr at the Rowett Research Institute,”Minerva1999372598010.1023/a:100475382547122029078
	    
	

5
	    LawrenceChristopherMedicine in the Making of Modern Britain: 1700–1920LondonRoutledge19947487 passim
	      
	    
	

6
	    *MRC Report 1928*–*29* (London: HMSO, 1930), p. 23. In fact, Lewis wrote most of the controversial pp. 22–29: see “Private and Confidential for Members of the MRC only. Draft for a Section of the Introduction to the Annual Report,” 26 December 1929, Lewis Papers, PP/LEW/D.4/1, Wellcome Library, London.
	

7
	    See esp. JewsonN. D.“The Disappearance of the Sick-Man from Medical Cosmology, 1770–1870,”Sociology1976102254410.1093/ije/dyp18019433521
	    
	

8
	    SmithDavidNicolsonMalcolm“The ‘Glasgow School’ of Paton, Findlay and Cathcart: Conservative Thought in Chemical Physiology, Nutrition and Public Health,”Soc. Stud. Sci.19891919523810.1177/03063128901900200111622464 SmithNicolson“Chemical Physiology versus Biochemistry, the Clinic versus the Laboratory: The Glaswegian Opposition to Edward Mellanby’s Theory of Rickets,”Proc. Roy. Coll. Physicians Edinburgh1989191516011612466 . Their analysis supported Jacyna, “Laboratory and the Clinic” (n. 1).
	

9
	    Smith and Nicolson, “Chemical Physiology” (n. 8), p. 51 (emphasis added).
	

10
	    Smith and Nicolson, “‘Glasgow School’” (n. 8), p. 196.
	

11
	    Ibid., p. 210.
	

12
	    Ibid., p. 197.
	

13
	    MorganNeil“The Strategy of Biological Research Programmes: Reassessing the ‘Dark Age’ of Biochemistry, 1910–1930,”Ann. Sci.19904713950 on p. 141 1161318410.1080/00033799000200171
	    
	

14
	    Ibid., p. 150.
	

15
	    Smith and Nicolson, “‘Glasgow School’” (n. 8), p. 208.
	

16
	    See HullAndrew“Hector’s House: Sir Hector Hetherington and the Academicization of Glasgow Medicine before the NHS,”Med. Hist.2001452074211373860PMC1044354
	    
	

17
	    Geison, “Social and Institutional Factors” (n. 2); Kohler, *From Medical Chemistry* (n. 2).
	

18
	    SturdySteve“Medical Chemistry and Clinical Medicine: Academics and the Scientisation of Medical Practice in Britain, 1900–1925,”Medicine and Change: Historical and Sociological Studies of Medical InnovationLöwyIlanaLondonJohn Libbey Eurotext199337193 on p. 372
	      
	    
	

19
	    Malcolm Nicolson and David Smith, “Science and Clinical Scepticism: The Case of Ralph Stockman and the Glasgow Medical Faculty” (paper presented at Science and Technology Dynamics Internal Progress Conference, Amsterdam, September 1997), p. 18. I am grateful to the authors for a copy of this paper.
	

20
	    PickstoneJohn V“Ways of Knowing: Towards a Historical Sociology of Science, Technology and Medicine,”Brit. J. Hist. Sci.19932643358 PickstoneWays of Knowing: A New History of Science, Technology and MedicineManchesterManchester University Press2000 . Pickstone also pioneered the importance of the local context: see, esp., Pickstone“Psychiatry in District General Hospitals: History, Contingency and Local Innovation in the Early Years of the National Health Service,”Medical Innovations in Historical PerspectivePickstoneLondonMacmillan199218599
	    
	

21
	    Valier, “Politics” (n. 4); Sturdy, “Political Economy” (n. 3).
	

22
	    Valier, “Politics” (n. 4), p. 98.
	

23
	    See PatonD. N.“The Relationship of Science and Medicine,”Edinburgh Medical J.192835111 , on p. 10, cited in Smith and Nicolson, “‘Glasgow School’” (n. 8), p. 206.
	

24
	    See Hull, “Hector’s House” (n. 16).
	

25
	    See Graham, “Formation” (n. 3); Weatherall, *Gentlemen* (n. 4).
	

26
	    See A. Flexner, *Medical Education in Europe*, Carnegie Foundation for the Advancement of Teaching, Bulletin no. 6 (New York: Carnegie Foundation, 1912), p. 131.
	

27
	    Paton was in charge of General or Experimental Physiology and Histology, and Cathcart of Physiological Chemistry.
	

28
	    See ThomsonR. Y.SmellieR. M. S. “Biochemistry,” Proc. Roy. Soc. Edinburgh Sect. B,1983842135 on p. 24 SimpsonElliottPeople Who Made Scottish Clinical BiochemistryGlasgowAssociation of Clinical Biochemists19952560
	    
	

29
	    The proximate cause for this was the 1922 MRC decision to discontinue the free supply of insulin: hospitals now needed somebody on staff to provide it, and to perform blood-sugar tests. Clinicians could not do the biochemistry, and the new biochemists had to be medically qualified to take blood. Thanks to Malcolm Nicolson for help on this point.
	

30
	    See Munk’s RollLondonRCPL196813031 VeederB. S.“Leonard Findlay,”Paediatric ProfilesVeederSt. LouisMosby195718992
	

31
	    See Archibald Young, *The Limitations of Surgery, Past and Present*, Presidential Address, Royal Medico-Chirurgical Society of Glasgow, 3 October 1924 (Glasgow: MacDougall, 1925), p. 31.
	

32
	    Sturdy and Cooter, “Science, Scientific Management” (n. 3). See also CooterRogerSurgery and Society in Peace and War: Orthopaedics and the Organization of Modern Medicine, 1880–1948ManchesterManchester University Press1993
	    
	

33
	    Smith, “Use of Team Work” (n. 4).
	

34
	    FindlayLeonard“Work Done by the Glasgow School on the Aetiology of Rickets,”Lancet192219982531 on p. 826
	      
	    
	

35
	    Royal Society of Medicine, Section of Medicine meeting, 28 October 1930, discussion on “Research in Medicine,”Lancet193021696667 on p. 966 (emphasis added)
	      
	    
	

36
	    RobertsonEdnaThe Yorkhill StoryGlasgowYorkhill Hospital Board of Management1972
	    
	

37
	    Donald MacAlister, Chairman of Governors and Patron of Hospital, moving adoption of Annual Report at Annual Meeting, 14 March 1923, RHSC *Annual Report* (1924), p. 11, Yorkhill Archives, Glasgow, YH 3/1/8.
	

38
	    See, e.g., ibid., pp. 11–12.
	

39
	    Directors’ Meeting, 14 July 1930, pp. 159–66, RHSC, *Minutes of Board of Management*, Yorkhill Archives, YH 1/2/10. For Findlay’s later career, see Lawrence Weaver, “From the Appliance of Science to an Alliance with Science: Leonard Findlay and the Making of a Modern Medical Unit” (paper delivered at Wellcome Trust Regional Forum on the History of Medicine in Scotland, Glasgow, October 2003).
	

40
	    See MacAlisterE.Sir Donald MacAlister of TarbetLondonMacmillan193534142 RHSC, *Minutes of Board of Management*, for the years 1918–27, Yorkhill Archives.
	

41
	    RHSC, *Annual Report* (1928), p. 21.
	

42
	    See Meeting of Directors, 9 January 1928, p. 216, in RHSC, *Minutes of Board of Management* (1925–29), Yorkhill Archives, YH 1/2/9 (hereafter RHSC *Minutes*), where this aim is explicitly stated in relation to the new University/Hospital posts.
	

43
	    Blacklock continued as Hospital Pathologist, a post he had held since 1924.
	

44
	    See Meetings of Directors on 10 January 1927, 8 August 1927, and 14 August 1927, esp. pp. 203–5, “Memorandum of Agreement between the University Court of the University of Glasgow and the Royal Hospital for Sick Children,” RHSC *Minutes*. Morris’s salary (£350 from the University as Lecturer; £200 from the Hospital as Clinical Biochemist) reflects his intended role.
	

45
	    Meeting of Directors, 9 January 1928, pp. 216–17, RHSC *Minutes*. Blacklock’s salary also reflected his role: £600 from the University for the Lectureship, and a further £350 from the Hospital as Pathologist (ibid., p. 217).
	

46
	    Directors’ Meetings on 9 January, 8 October, 12 November 1928, RHSC *Minutes*.
	

47
	    Directors’ Meeting, 10 December 1928, supplement, “Memorandum by the Directors, November 1928,” pp. 336–46, on p. 338, RHSC *Minutes*.
	

48
	    Ibid., pp. 342–43 (emphasis added).
	

49
	    “University of Glasgow Regius Chair of Materia Medica. Application with Testimonials in Favour of Noah Morris,” 1937, Glasgow University Archives and Business Records Centre, HP, DC 8/888, pp. 1–2.
	

50
	    See *MRC Reports*, 1928–48, passim.
	

51
	    MorrisNoah“The Cause of Increased Electrical Excitability,”Glasgow Med. J.19229832937PMC593616830435624 , on pp. 336–37, citing FindlayL.FergusonM.A Study of Social Factors in the Causation of Rickets MRC Special Report Series, no. 20 LondonMRC1918
	  
	

52
	  See Noah Morris, “Metabolic Studies in Disorders of Acid-Base Equilibrium of Infancy and Childhood” (D.Sc. thesis, University of Glasgow, 1935), acknowledgments section, where he thanks them for access to their patients.
	

53
	  MorrisNoahGrahamStanley“The Regulation of the Acid-Base Balance of the Body,”Glasgow Med. J.192810923960 on p. 247 (emphasis added) PMC597031030438298
	  
	

54
	  MorrisNoahGrahamStanley“Observations on the Action of Acetone,”Arch. Dis. Childh.192722121910.1136/adc.2.10.212PMC197497421031698 MorrisGraham“Regulation of the Acid-Base Balance of the Body,”Trans. Roy. Med.-Chirurg. Soc. Glasgow1927–282297118 MorrisGraham“Carbon Dioxide Dissociation Curve of Blood in Infancy and Childhood,”Arch. Dis. Childh.1929429930510.1136/adc.4.23.299PMC197507921031776 MorrisGraham“Changes in Blood Chemistry in Congenital Hypertrophic Pyloric Stenosis and Their Clinical Significance,”ibid.3354610.1136/adc.4.23.335PMC197508021031782 GrahamMorris“Changes in Blood Density in Congenital Hypertrophic Pyloric Stenosis and Their Clinical Significance,”ibid.193052072810.1136/adc.4.23.335PMC197508021031782 MorrisGraham“The Theoretical Carbon Dioxide Dissociation Curve in Acid-Base Disturbance of Childhood,”Brit. J. Exp. Path.19301132228 MorrisGraham“Observations on Chloride Metabolism in Congenital Pyloric Stenosis,”Arch. Dis. Childh.19316273610.1136/adc.6.31.27PMC197514121031835
	  
	

55
	  Morris and Graham, “Regulation of the Acid-Base Balance” (n. 53), p. 257.
	

56
	  Ibid.
	

57
	  Ibid. (emphasis added).
	

58
	  Lawrence, “Incommunicable Knowledge” (n. 1), p. 515.
	

59
	  Morris and Graham, “Regulation of the Acid-Base Balance” (n. 53), p. 259.
	

60
	  GrahamStanleyMorrisNoahAcidosis and AlkalosisEdinburghLivingstone1933vii
	  
	

61
	  Morris, “Metabolic Studies” (n. 52), pp. 1–2.
	

62
	  On triangulation, see StarSusan Leigh“Triangulating Clinical and Basic Research: British Localizationists, 1870–1906,”Hist. Sci.19862429481162092210.1177/007327538602400102
	  
	

63
	  Morris, “Metabolic Studies” (n. 52), pp. 11–12.
	

64
	  PatonD. N.“Physiology in the Study of Disease,”Physiology and National NeedsHalliburtonW. D.LondonConstable191975101 on p. 80
	    
	  
	

65
	  Ibid., pp. 81–82, 99.
	

66
	  This scientific holism is very clearly expressed in the introduction to Morris’s D.Sc. thesis, “Metabolic Studies” (n. 52), pp. 2–5. See also Noah Morris, “The History of Therapeutics,”Proc. Roy. Phil. Soc. Glasgow1944–45691334 on pp. 27–29
	    
	  
	

67
	  See FindlayL.“The Study and Teaching of Paediatrics. Abstract of a Lecture to the Belgrade Faculty of Medicine and Medical Association, Yugoslavia,”Brit. Med. J.December 192928118588
	  
	

68
	  MorrisNoah“Prolegomena to the Study of Therapeutics,”Glasgow Med. J.1937 7th ser. 128413751 on p. 138 PMC598140330439118
	

69
	Morris, “Metabolic Studies” (n. 52), p. 4.

70
	Morris, “History of Therapeutics” (n. 66), p. 33.

71
	MorrisNoah“The Significance of Plasma Phosphatase in Bone Disease and Jaundice” (summary of a paper given to the Royal Medico-Chirurgical Society of Glasgow, 4 December 1936),Glasgow Med. J.1937, 7th ser1272526 MorrisNoahPedenOlive D.“Plasma Phosphatase in Disease: A Review,”Quart. J. Med.n.s., 1937621130 MorrisNoahStevensonM. M.PedenO. D.SmallJ. M. P.“Significance of Plasma Phosphatase in the Diagnosis and Prognosis of Rickets,”Arch. Dis. Childh.193712455810.1136/adc.12.67.45PMC197549421032054
	

72
	The names of both the department and the chair were changed to Biochemistry in 1958: see Thomson and Smellie, “Biochemistry” (n. 28).

73
	Morris, “Significance of Plasma Phosphatase in Bone Disease and Jaundice” (n. 71), pp. 25–26 (emphasis added).

74
	Morris, Stevenson, et al., “Significance of Plasma Phosphatase” (n. 71), p. 45 (emphasis added).

75
	Morris and Peden, “Plasma Phosphatase in Disease” (n. 71), p. 221 (emphasis added).

76
	See Findlay’s remarks at the Royal Society of Medicine in 1930, as reported in Proc. Roy. Soc. Med.1930–3124115268 on pp. 166–67
	  
	

77
	Morris, “Prolegomena” (n. 68), p. 138 (emphasis added).

